# Performance of Syphilis Sentinel Surveillance in the context of endemic Treponematoses: experience from Ghana

**DOI:** 10.1186/s12879-016-2085-y

**Published:** 2016-12-09

**Authors:** Edward Tieru Dassah, Yaw Adu-Sarkodie, Philippe Mayaud

**Affiliations:** 1 Department of Obstetrics and Gynaecology, Komfo Anokye Teaching Hospital, P. O. Box KS 1934, Kumasi, Ghana; 2 School of Public Health, Kwame Nkrumah University of Science and Technology, Kumasi, Ghana; 3 School of Medical Sciences, Kwame Nkrumah University of Science and Technology, Kumasi, Ghana; 4 London School of Hygiene and Tropical Medicine, London, UK

**Keywords:** Syphilis surveillance, Syphilis seroprevalence, Treponemal tests, Non-treponemal tests, Yaws, HIV sentinel survey, Ghana

## Abstract

**Background:**

Use of treponemal tests to screen for syphilis (caused by *Treponema pallidum pallidum*) poses challenges with infection status classification, especially in settings where other treponemal infections are endemic. This study aimed to determine the validity of the syphilis surveillance testing strategy implemented since 2004 using two treponemal tests, and estimate the seroprevalence of active syphilis infection in Ghana where yaws (caused by *Treponema pallidum pertenue)* is endemic.

**Methods:**

We retested sera from the 2007 HIV sentinel survey (HSS) using a traditional algorithm, quantitative rapid plasma reagin test followed by qualitative *Treponema pallidum* haemagglutination assay. The adjusted seroprevalence of active syphilis was calculated by applying the proportions of active syphilis within identified categories of HSS samples during the retesting, to the entire population of HSS samples. The 95% confidence intervals (CIs) were calculated for each proportion, and the *t*-test was used to assess differences in proportions.

**Results:**

Of 2,214 samples that were retested, 203 (9.2%) had active syphilis infection, 21 (0.9%) were biological false reactions, 640 (28.9%) were past or treated syphilis infections, and 1,350 (61%) were uninfected. The current syphilis testing strategy overestimated the seroprevalence of active syphilis infection by a third (HSS versus traditional algorithm: 6.0% (95% CI: 5.6–6.3) vs. 4.5% (95% CI: 4.2–4.8); *p <* 0.001), and had low positive predictive value (16.8%) for detecting active syphilis infection. More than half (51.9%) of HSS syphilis positive cases were actually past/treated treponemal infections, possibly previous exposure to yaws.

**Conclusion:**

There is an urgent need to review the current syphilis sentinel surveillance testing strategy in Ghana in the context of concurrent endemic treponematoses, to better inform policy.

## Background

Surveillance of syphilis seroprevalence is a key component of the World Health Organization’s (WHO) strategy for the global elimination of congenital syphilis [1]. Syphilis seroprevalence surveillance among pregnant women has been conducted as part of HIV sentinel survey (HSS) in many countries, including Ghana [2], and is a proxy indicator for monitoring the prevalence of sexually transmitted infections (STIs) in the general population [2, 3]. Serological testing for syphilis involves the detection of two types of antibodies, non-treponemal and treponemal antibodies. Non-treponemal antibodies become reactive during the inflammatory phase caused by acute syphilis and non-reactive (serorevert) after successful treatment or in long-standing/late stages of treponemal infections, while the specific treponemal antibodies rise during the acute phase and usually remain detectable for life, even after successful treatment [3, 4]. Therefore, traditionally, serologic testing for syphilis involves screening with a non-treponemal test such as rapid plasma reagin (RPR) to identify persons with possible untreated infections, followed by confirmation using a treponemal test (e.g. *Treponema pallidum* haemagglutination assay [TPHA]) [3, 4]. For purposes of high throughput testing, some laboratories have adopted a reverse screening sequence; screening with an automated treponemal test first followed by confirmation with a non-treponemal test [4]. This reverse algorithm still requires the use of a non-treponemal test to identify active infections [4].

For reasons of performance and convenience, the HSS resorted to the use of two treponemal tests for syphilis sentinel surveillance testing in Ghana since 2004 [5]. The use of a treponemal test for syphilis (caused by *Treponema pallidum* subspecies *pallidum*) screening poses challenges with classification of infection status [4], especially in settings where other treponemal infections including yaws (caused by *Treponema pallidum* subspecies *pertenue*) are endemic [3]. The aims of this study were (1) to determine the validity of the current syphilis surveillance testing strategy in Ghana, which has been implemented since 2004, in comparison to the traditional WHO-recommended algorithm (i.e., RPR followed by TPHA) as the gold standard [6]; and (2) to estimate the seroprevalence of active maternal syphilis by retesting archived sera from the 2007 HSS in the context of endemic yaws. We hypothesized that syphilis sentinel surveillance using two treponemal tests (Determine syphilis TP [DS] and TPHA tests) overestimates the seroprevalence of active maternal syphilis in Ghana.

## Methods

### Study context and setting

Yaws and syphilis are the two treponemal infections of public health importance in Ghana. Yaws is transmitted by direct skin-to-skin contact mainly among children under 15 years of age. Congenital infections do not occur, possibly because active yaws is rare among women of childbearing age [7]. Yaws has remained a public health problem in Ghana over the past five decades, although the prevalence varies between regions [8, 9]. Despite earlier success of eradication campaigns with mass penicillin treatment in the 1950s and 1960s [8], the disease still occurs in all 10 regions of the country [9]. The highest incidence has been reported in warm regions with high levels of humidity and rainfall, especially the Eastern, Central, Western, Ashanti and Volta regions, while the three northern regions (Northern, Upper East and Upper West), which are predominantly warm and dry, have the lowest incidence rates in the country [9].

Syphilis is a global health problem but has not been a major clinical problem in Ghana since the 1960s [1, 10]. While clinicians in STI clinics and general hospital settings report attending to cases of the major STIs, they hardly appear to see any cases of syphilis [10]. The very low occurrence of clinically recognisable syphilis has been attributed to the low prevalence of infection, which resulted in part from the mass penicillin treatment campaigns against yaws in the 1950s and 1960s [8, 10].

The national HSS has been conducted in 40 sites involving over 18,000 antenatal attendees across the 10 regions of the country since 2005. The HSS data has reported highly variable “syphilis” seroprevalence rates ranging from 0 to 33.9% [2], with a reported mean syphilis seroprevalence of <0.5% (range 0–0.8%) prior to 2004 [1], climbing to 6.6% in 2004, and ranging from 5.5 to 6.5% thereafter [2, 5] after a change in the testing algorithm. The HSS syphilis testing strategy departed from the traditional WHO-recommended algorithm of screening with RPR followed by TPHA confirmation [6], to the use of a treponemal test (DS) for screening and maintaining the “confirmatory” test as TPHA, thereby using two treponemal tests sequentially [2, 5, 11]. The switch of screening test from RPR to DS was implemented for reasons of performance (higher sensitivity) and practicality [5].

In 2003, the reported HSS regional “syphilis” seroprevalence rates were much lower than those of HIV seroprevalence [11]. Since 2004, the Central, Eastern and Western regions have had the highest regional “syphilis” seroprevalence rates which are about 1.5 to 11 fold higher than the corresponding HIV seroprevalence. However, the three northern regions with the lowest “syphilis” seroprevalence have rates similar to those of HIV [2, 5]. Notably also, “syphilis” seroprevalence among 15–19 year olds have become much higher (1.5 to 6 fold higher rates) than HIV since 2004, while the converse was the case prior to 2004 [2, 5].

### 2007 HSS and selection of samples for retesting

The 2007 HSS involved 18,366 participants between the ages of 15 and 49 years. Almost all (18,048, 98.3%) were pregnant women attending antenatal services whilst the remainder (318, 1.7%) were STI clinic attenders. All samples were initially tested at the various sentinel sites with DS: DS-positive samples were further tested centrally at the National Public Health and Reference Laboratory with TPHA, whilst no further testing was done for DS-negative samples. Of the 18,366 samples, 1,097 (6.0%) were positive for both DS and TPHA and classified as “syphilis positive” After the survey, all samples were stored at the National Public Health and Reference Laboratory without any personal identifiers.

The list of all samples from the 2007 HSS was obtained from the National AIDS/STI Control Programme (NACP) for review and selection of samples for retesting. The samples comprised of 1,294 (7.0%) DS-positive, of which 1,097 (84.7%) were positive for TPHA, 135 (10.5%) negative for TPHA, and 62 (4.8%) whose TPHA results were not available. Determine Syphilis TP results for 31 TPHA-positive samples were also not available, and these samples were excluded from the subsequent analysis to estimate the seroprevalence of active syphilis.

Sample selection for the retesting was weighted according to the number of DS-positives in each site; for every DS-positive sample the next consecutive DS-negative sample (from the same site) was also selected. A total of 2,214 samples (12.1% of all HSS samples) were retrieved for retesting, 1,096 DS-positive and 1,118 DS-negative samples (Table 1).

**Table 1:** Ghana HIV Sentinel Survey (HSS) 2007 syphilis test results of samples selected for syphilis serology retesting

Determine Syphilis TP results	TPHA results			
	Positive	Negative	Not done	Total
Positive	1,001	59	36	1,096
Negative	0	0	1,118	1,118
Total	1,001	59	1,154	2,214

*TPHA Treponema pallidum* haemagglutination assay

### Retesting of sera and interpretation of test results

Selected serum samples were transported on dry ice to the Department of Clinical Microbiology, School of Medical Sciences, Kwame Nkrumah University of Science and Technology (KNUST) and Komfo Anokye Teaching Hospital for retesting. All samples were retested using a quantitative RPR test (Omega Diagnostics, Alva Scotland, UK) and a qualitative TPHA test (Omega Diagnostics, Alva Scotland, UK) according to the manufacturers’ recommendations. We did not have an independent treponemal assay to perform fluorescent treponemal antibody absorption (FTA-ABS) test for the few RPR-positive/TPHA-negative samples, as usually recommended [3, 12]; instead, these samples were retested with DS.

Samples which were positive by both RPR and TPHA were classified as having “active syphilis”. These were further classified as high titre active syphilis if the RPR titre was ≥1:8, or low titre active syphilis if the RPR titre was <1:8. Samples which were positive for RPR and negative for TPHA were retested with DS and classified as active syphilis if the DS was positive and biological false reaction if DS was negative. Samples which were negative for RPR and positive for TPHA were classified as past or treated syphilis infection. Samples which were negative for both RPR and TPHA were classified as syphilis-uninfected [12].

### Sample size estimation

A sample of 2000 DS tested samples (1000 positives and 1000 negatives) from the 2007 HSS was required to estimate a positive predictive value (PPV) or negative predictive value (NPV) of 20% (95% confidence interval [CI]: 17.6–22.6) and 90% (95% CI: 88.0–91.8), respectively.

### Statistical analyses

**Fig. 1:**
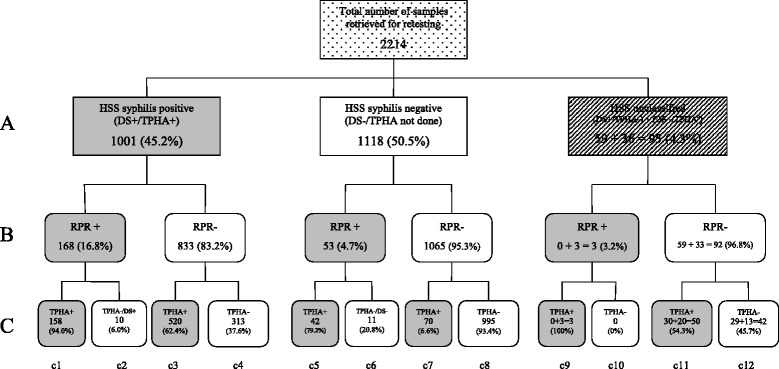
Serology results of selected samples from the 2007 HIV Sentinel Survey (HSS) and retesting with RPR and TPHA. DS-Determine syphilis TP; TPHA- *Treponema pallidum* haemagglutination assay; TPHA^0^-TPHA result not available; RPR-rapid plasma reagin; Level *A* = HSS results of selected samples; Levels *B* & *C* = Results of retesting with RPR, TPHA and DS

The adjusted seroprevalence of active syphilis was calculated by applying the seroprevalence of active syphilis within four categories of HSS samples (DS+/TPHA+, DS+/TPHA−, DS+/TPHA not available, and DS-/TPHA not done; Fig. 1) that were identified during retesting, to their respective categories in the entire population of 18,366 HSS samples. The 95% CIs were calculated for each proportion, and the *t*-test was used to assess differences in proportions.

## Results

The results of the selected HSS samples and the retesting with RPR and TPHA are summarised in Fig. 1 and Table 2.

**Table 2:** Comparing results of HIV Sentinel Survey (HSS) and conventional syphilis testing strategies using selected archived sera from 2007 HSS in Ghana

Results (and interpretation) of RPR/TPHA retesting	Results of HSS testing strategy *n* (%)				
	DS+/TPHA+	DS−/TPHA−	DS+/TPHA−	DS+/TPHA^0^	Total
RPR+/TPHA+ (Active infection)	168 (16.8)^a^	42 (3.8)	0 (0)	3 (8.3)	213 (9.6)
RPR+/TPHA−(Biological false positive reaction)	0 (0)^a^	11 (1.0)	0 (0)	0 (0)	11 (0.5)
RPR−/TPHA+ (Past/treated infection)	520 (51.9)	70 (6.2)	30 (50.8)	20 (55.6)	640 (28.9)
RPR−/TPHA−(Uninfected)	313 (31.3)	995 (89.0)	29 (49.2)	13 (36.1)	1,350 (61.0)
Total	1,001 (100)	1,118 (100)	59 (100)	36 (100)	2,214 (100)

*RPR* rapid plasma reagin, *TPHA Treponema pallidum* haemagglutination assay, *TPHA* ^0^− *TPHA* results not available, *DS* determine syphilis TP, ^a^Includes 10 RPR+/TPHA − samples which were DS+; Excludes 10 RPR+/TPHA − samples which were DS+

Of the 2,214 retested samples, 203 (9.2%; c1, c5 and c9 cells in Fig. 1) were positive by both RPR and TPHA (active syphilis infection), 21 (0.9%; c2, c6 and c10 cells) samples were RPR-positive and TPHA-negative (biological false reactions), 640 (28.9%; c3, c7 and c11 cells) samples were RPR-negative and TPHA-positive (past or treated syphilis infection), and 1,350 (61%; c4, c8 and c12 cells) samples were negative by RPR and TPHA (uninfected). When the 21 RPR+/TPHA- samples were retested with DS, 10 were positive for DS and classified as having active syphilis (c2 cell), while the remaining 11 were negative for DS and classified as biological false reactions (c6 cell). Determine Syphilis TP results for those 21 samples were the same as those initially obtained during the HSS (see corresponding A cells for c2 and c6 cells). Overall, 213 samples were classified as having active syphilis (Table 2), 145 (68.1%) had low titre (<1:8) active syphilis, while the remaining 68 (31.9%) had high titre (≥1:8) active syphilis (data not shown). About one third (323/1001; c2 and c4 cells) of samples that were TPHA-positive and half (30/59; cell c11) of samples that were TPHA-negative during the HSS were found to have the opposite result (i.e. being TPHA-negative and TPHA-positive respectively), during retesting.

Results of the selected samples using HSS testing strategy (two treponemal tests) and conventional testing algorithm (RPR/TPHA) are compared in Fig. 1 and Table 3. Of the 1,001 samples that were classified as cases of “active syphilis infection” by the HSS testing strategy, only 168 (16.8%) (including the 10 RPR+/TPHA- samples which were DS+, c2 cell) had active syphilis infection using the traditional algorithm; actually, more than half (520 [51.9%], c3 cell) of HSS active syphilis samples were cases of past or treated syphilis or treponemal infections. Most samples (995 [89%], c8 cell) classified as not having any syphilis infection were also found to be negative for syphilis using the conventional algorithm. However, 42 (3.8%, c5 cell) of samples considered to be negative for syphilis during the HSS were found to have active syphilis infection using the conventional strategy. Three (8.3%) samples that were positive for DS but had no available TPHA results from the HSS were found to have active syphilis during retesting (c9 cell). None of the DS-positive TPHA-negative samples was positive for syphilis during retesting. Using data from the 2007 HSS (Table 3), the seroprevalence of “active syphilis” infection (1,097/18,366) was 6.0% (95% CI: 5.6–6.3). Using data in Table 3 for seropositivity rate in each sub-category of results for the HSS and applied to the category distribution of the entire HSS panel (and excluding 31 samples for which DS results were not available) the seroprevalence of active syphilis using the conventional algorithm was estimated as (818.5/18,335) or 4.5% (95% CI: 4.2–4.8). As expected, the use of two treponemal tests significantly overestimated the seroprevalence of active syphilis in Ghana by one-third compared to RPR/TPHA as the gold standard algorithm (6.0% vs. 4.5%; *p <* 0.001).

**Table 3:** Summary of 2007 HIV Sentinel Survey (HSS) syphilis test results and results of samples selected for retesting

Categories of HSS results	DS+/TPHA+	DS−/TPHA^0^	DS+/TPHA−	DS+/TPHA^0^	^a^DS^0^/TPHA+	Total
Number of all HSS samples	1,097	17,041	135	62	31	18,366
Number of selected samples	1,001	1,118	59	36	0	2,214
Number of RPR+/TPHA+ cases	158	42	0	3	0	203
Estimated number of active syphilis cases	173.15	640.18	0	5.17	NS	818.50

*DS* Determine syphilis TP, *TPHA Treponema pallidum* haemagglutination assay, *RPR* Rapid plasma reagin, *TPHA* ^0^ − *TPHA* result not available, *DS* ^0^ DS result not available, *NS* samples not selected for retesting, ^a^DS^0^/TPHA+ samples (*n* = 31) were excluded from the final analysis. Therefore, denominator for the overall estimate was (18,366–31) =18,335

Note: Only DS+/TPHA+ samples were regarded as “syphilis” seropositive samples (more accurately, treponemal seropositives) during the sentinel survey; all other samples were considered to be negative

## Discussion

The seroprevalence of “active syphilis” in Ghana had increased dramatically since 2005 when the NACP adopted a strategy of using two sequential treponemal tests for syphilis sentinel surveillance. Serological evidence from retesting using the traditional algorithm and the knowledge of regional epidemiology of yaws in Ghana suggest that the use of two treponemal tests may be overestimating the seroprevalence of active syphilis.

Most authorities recommend screening for syphilis with a non-treponemal test and subsequent confirmation of positive results with a specific treponemal test [3, 4, 6]. This traditional algorithm avoids problems with classification of infection status when a reverse algorithm (screening with a treponemal test followed by a non-treponemal test) or using two treponemal tests for the diagnosis of active syphilis is used [4]. The choice of a suitable testing algorithm will also depend on the epidemiology of treponemal infections in the area. Yaws has been endemic in Ghana over the past five decades [8, 9], while syphilis had been relatively rare [10] until recent HSS data indicated dramatic increases in “syphilis” (or more accurately, “treponemal infection”) seroprevalence following a change in the testing algorithm (from using RPR/TPHA to the use of two treponemal tests) [1, 2, 5].

Not surprisingly, the current HSS testing strategy has been overestimating the seroprevalence of active maternal syphilis by a third since 2004. Less than one fifth of samples identified as having “active syphilis infection” by the HSS testing strategy were classified as “active syphilis infection” by the traditional algorithm indicating that the HSS strategy has a low PPV (16.8%) for detecting active syphilis infection. As expected, over half of samples considered to have “active syphilis infection” by the HSS strategy were classified as past or treated infections using the traditional algorithm. Our results are in agreement with those of a recent study conducted among 200 blood donors in Kumasi; using two treponemal tests (an enzyme immunoassay and TPHA) the seroprevalence of syphilis was reported to be 8% while the seroprevalence of active syphilis (using RPR and either treponemal test) was much lower, 3.5% [13]. Treponemal tests reactivity persists over a lifetime. Therefore, reactive treponemal tests alone are neither indicative of active infection nor of the need for treatment but simply reflect the cumulative incidence of treponemal infections over time [3, 4, 6, 14]. This is however a function of survival of infected individuals, so seroprevalence may not consistently rise with age. On the other hand, non-treponemal tests can be non-reactive in the very early stages of syphilis infection and during late stages of the infection, and usually serorevert (become non-reactive) after successful treatment [4]. Therefore, samples which were classified as having past or treated syphilis infection (RPR−/TPHA+) could have had either early, past or treated syphilis infection. However, given the epidemiology of yaws and syphilis in Ghana [1, 8–10], it is more likely that most of these samples were from individuals who were previously exposed to yaws rather than syphilis. It is worth noting that, whatever the true classification of these past or treated treponemal infections may be (i.e. whether past or treated syphilis or yaws), they are not known to be associated with adverse pregnancy outcomes [12, 15]. Our suspicion that most past treponemal infections (RPR−/TPHA+) were more likely to have been due to previous exposure to yaws rather than syphilis is strengthened by the following ecological observations:

i. The pattern of regional distribution of yaws seems to reflect regional HSS syphilis seropositivity in most instances, suggesting that treponemal test seropositivity may be due to previous exposure to yaws, since clinically recognisable syphilis has been relatively rare in Ghana.
ii. In the younger age groups of 15–19 years treponemal tests seroprevalence generally far exceeds that of HIV (since 2004) suggesting a non-venereal treponemal infection rather than syphilis. This is consistent with ongoing and not too distant yaws transmission in these communities [14]. It is unlikely that these adolescents acquired syphilis congenitally, as they would not have survived without any treatment.

Generally, in settings of high treponemal infections (including yaws), treponemal tests alone tend to overestimate the seroprevalence of active syphilis infection, and are best used in conjunction with non-treponemal tests [3, 6, 16]. In fact, WHO recommends the use of a non-treponemal test for screening in such high prevalence settings [6]. Where treponemal tests are used for screening, it is recommended that seropositive samples should be subsequently tested with quantitative non-treponemal tests to determine infection status (past or active infection) [3, 16]. The findings of this study notwithstanding, it must be emphasized that pregnant women who test positive for syphilis using treponemal point-of-care tests during antenatal care must be treated on the same visit rather requesting further testing with non-treponemal tests to avoid delays and missed opportunities: the benefits of early treatment far outweigh the risks of overtreatment (which are rather rare) [3, 16].

The study had some limitations. First, because of the matching system used in selection, negative samples from sites with high HSS syphilis positivity rates were more likely to be selected, and indeed samples from one site out of 40 which did not have positive samples ended up not being included. Second, the true syphilis infection status of 21 RPR+/TPHA- samples could not be determined because we could not use a tie-breaker test such as the FTA-ABS. However, the overall adjusted prevalence of active syphilis was unlikely to be affected due to the relatively small number of such samples (0.9% of total). Third, 96 (8.8%) of HSS syphilis positive samples were not available for retesting, leading to slight inaccuracy of results. It is possible that some samples were used up during the HSS testing. Fourth, repeated freeze-thaw cycles may have affected the sensitivity of our results. However, the loss in sensitivity is expected to be marginal as the necessary precautions were taken to minimise the number of freeze-thaw cycles.

## Conclusion

The nationally adopted HSS syphilis testing strategy of using two sequential treponemal tests for convenience reasons is particularly not suited for detecting active syphilis infection in Ghana, thereby undermining its informative purpose. There is an urgent need to review the strategy for syphilis surveillance to obtain accurate data that are necessary to inform policy and track impact of interventions.

## Abbreviations

CI: Confidence interval
DS: Determine syphilis TP
FTA-ABS: Fluorescent treponemal antibody absorption
HSS: HIV sentinel survey
KNUST: Kwame Nkrumah University of science and technology
NACP: National AIDS/STI Control Programme
NPV: Negative predictive value
PPV: Positive predictive value
RPR: Rapid plasma reagin
STI: Sexually transmitted infections
TPHA: *Treponema pallidum* haemagglutination assay
WHO: World Health Organization
